# Role of circulating tumor DNA to help decision-making in hepatocellular carcinoma

**DOI:** 10.18632/oncoscience.446

**Published:** 2018-08-22

**Authors:** Johann von Felden, Amanda J. Craig, Augusto Villanueva

**Affiliations:** Division of Liver Diseases, Division of Hematology and Medical Oncology, Department of Medicine, Liver Cancer Program, Tisch Cancer Institute, Graduate School of Biomedical Sciences, Icahn School of Medicine at Mount Sinai, New York, NY 10029 USA

**Keywords:** liver cancer, next-generation sequencing, liquid biopsy, biomarker, prediction

Liver cancer is the second-most common cause of cancer-related mortality worldwide, and hepatocellular carcinoma (HCC) accounts for 90% of all cases. According to the current EASL guidelines, one of the unmet needs in HCC research is to develop new tools for early detection including the assessment of liquid biopsy [1]. Conceptually, liquid biopsy enables non-invasive analysis of tumor molecular alterations through isolation of tumor components that are released to the bloodstream or other body fluids. This includes circulating tumor DNA ctDNA) or RNA, circulating tumor cells (CTC), and exosomes. The majority of studies evaluated mutations of ctDNA to detect minimal residual disease, as diagnostic markers or to predict response to therapy. Most of them used either next generation sequencing (NGS) or digital PCR to map genetic alterations in ctDNA. While the latter is based on high sensitivity analysis of single genomic loci, NGS-based methods are able to interrogate the entire DNA sequence of a larger panel of genes, including identification of novel genetic or epigenetic alterations. This seems particularly useful when it comes to mutation profiling and monitoring tumor clonal evolution (i.e. emergence of new mutations). However, the optimization of NGS-based approaches are still more cost-, time-, and resource-consuming compared to digital PCR.

A multivariate blood test (i.e., cancerSEEK), incorporating ctDNA and established tumor protein markers, was recently reported for early detection of cancer [2]. Using 1,000 patients with different tumors, this test yielded a sensitivity from 69 to 98%, and a specificity up to 99% [2], suggesting that ctDNA may indeed have a role in cancer early detection. This study only included 39 HCC patients, mostly at intermediate or advanced stages, which limits its extrapolation to HCC patients at early stages, who represent the ideal target population for early detection programs [2]. More recently, we have shown the feasibility of mutation detection by ultra-deep targeted ctDNA sequencing in early stage HCC along with correlation of corresponding multiregional tissue samples. With a targeted panel of 58 frequently mutated and/or actionable genes in HCC, we were able to identify 70% of the ones present in paired tissue of the same patient [3].

A number of studies have explored how ctDNA could be used as a prognostic and predictive biomarker, mainly as a tool to identify minimal residual disease [4,5]. Assessment of ctDNA after radiotherapy in lung cancer was highly specific for the detection of tumor recurrence and correlated to worse overall survival [4]. Additionally, ctDNA detection outperformed conventional imaging by identifying recurrence 5.2 months earlier [4]. Similar results have been reported in 159 patients with locally advanced colorectal cancer, treated in the context of a clinical trial [5]. Postoperative detection of ctDNA correlated with recurrence irrespective of adjuvant chemotherapy and remained an independent predictor of recurrence-free survival after adjustment for established risk factors [5]. Collectively, these data further suggest the potential clinical utility of ctDNA to identify patients with residual disease after curative therapies.

**Figure 1:**
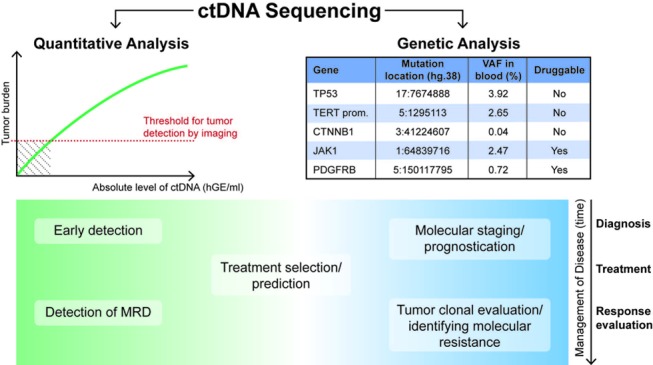
Summary of potential clinical applications of ctDNA sequencing throughout different stages of cancer management Quantitative analysis of ctDNA is based on a positive correlation of ctDNA concentration (e.g. haploid genome equivalents per mL, hGE/mL) and (absolute or metabolic) tumor burden [4,7], and thus potentially able to overcome a limiting detection threshold of imaging for very small tumors (hatched area). For example, in the setting of detection of minimal residual disease (MRD) after surgical and/or locoregional therapies. In contrast, analysis of specific genetic (e.g. mutations, see table) [3] or epigenetic (e.g. DNA methylation) aberrations could allow to identify targets for therapies and monitor how clonal composition evolves over time and upon exposures to treatments [6,7]. This might facilitate real time identification of molecular mechanisms of tumor resistance.

Besides allowing the quantitation of ctDNA, NGS enables identifying specific genomic alterations, which expands its applications to predictive biomarkers, monitoring of tumor clonal composition and identifying mechanisms of treatment resistance to systemic therapies (Figure 1). This is particularly interesting considering the difficulties to access tumor tissue in HCC patients, and the inherited constraints of sequential tissue biopsies. A phase 2 trial detected truncating mutations in DNA repair genes (e.g., *BRCA2* or *ATM*), and somatic alterations in *TP53* in ctDNA of patients with metastatic prostate cancer. These alterations were predictive of response in treatment- naïve patients receiving androgen receptor (AR)-directed therapies [6]. The ability to trace clonal tumor evolution by ctDNA monitoring has been demonstrated recently in early stage lung cancer [7]. Moreover, this study confirmed the power of ctDNA to predict treatment response [7]. As the number of effective systemic agents in HCC increases (i.e., sorafenib, lenvatinib, regorafenib, cabozantinib, ramucirumab), it will be paramount to maximize treatment response by selecting the best candidates for each therapy. Tumor characteristics (e.g. tumor burden) and technical considerations (e.g. targeted approach versus whole exome sequencing, data processing) are likely influencing ctDNA detection rates [3-7]. Numerous advancements (e.g. molecular barcoding, *in silico* error suppression, etc.) have helped to handle sequencing errors and improve sensitivity and specificity of ctDNA [4]. Nevertheless, analytical validity remains a concern with this technology, particularly after a study that compared the performance of two commercially available ctDNA detection assays (Guardant360 and PlasmaSELECT) in 40 patients with metastatic prostate cancer. Considering genes only targeted by both assays and after correcting for differences in exome coverage, only 12 of 40 patients (30%) had complete congruence between the two tests (identical or no mutations detected in both tests), while sixteen patients (40%) had incongruent results [8].

In summary, NGS of ctDNA has been increasingly tested in translational and clinical trial settings, particularly assessing treatment response and tumor clonal evolution, and may represent a powerful tool to help unmet challenges in clinical management of HCC. However, improved sensitivity and cross-validation among ctDNA assays will be required before we fully understand the real possibilities of this new technology.
